# Genetic management on the brink of extinction: sequencing microsatellites does not improve estimates of inbreeding in wild and captive Vancouver Island marmots (*Marmota vancouverensis*)

**DOI:** 10.1007/s10592-022-01429-7

**Published:** 2022-01-16

**Authors:** Kimberley G. Barrett, Geneviève Amaral, Melanie Elphinstone, Malcolm L. McAdie, Corey S. Davis, Jasmine K. Janes, John Carnio, Axel Moehrenschlager, Jamieson C. Gorrell

**Affiliations:** 1 Biology Department, Vancouver Island University, Nanaimo, BC V9R 5S5 Canada; 2 Marmot Recovery Foundation, Nanaimo, BC V9R 6X9 Canada; 3 Present Address: Department of Biological Sciences, University of Alberta, Edmonton, AB T6G 2R3 Canada; 4 School of Environmental and Rural Science, University of New England, Armidale, NSW 2351 Australia; 5 Wilder Institute Calgary Zoo, Calgary, AB T2E 7V6 Canada; 6 IUCN Species Survival Commission, Conservation Translocation Specialist Group, Calgary, AB Canada; 7 Present Address: Island Medical Program, University of Victoria, 3800 Finnerty Road, Victoria, BC V8P 5C2 Canada

**Keywords:** Captive-breeding, Conservation translocation, Genetic diversity, High-throughput amplicon sequencing, Relatedness, Studbook

## Abstract

Captive breeding is often a last resort management option in the conservation of endangered species which can in turn lead to increased risk of inbreeding depression and loss of genetic diversity. Thus, recording breeding events via studbook for the purpose of estimating relatedness, and facilitating mating pair selection to minimize inbreeding, is common practice. However, as founder relatedness is often unknown, loss of genetic variation and inbreeding cannot be entirely avoided. Molecular genotyping is slowly being adopted in captive breeding programs, however achieving sufficient resolution can be challenging in small, low diversity, populations. Here, we evaluate the success of the Vancouver Island marmot (*Marmota vancouverensis*; VIM; among the worlds most endangered mammals) captive breeding program in preventing inbreeding and maintaining genetic diversity. We explored the use of high-throughput amplicon sequencing of microsatellite regions to assay greater genetic variation in both captive and wild populations than traditional length-based fragment analysis. Contrary to other studies, this method did not considerably increase diversity estimates, suggesting: (1) that the technique does not universally improve resolution, and (2) VIM have exceedingly low diversity. Studbook estimates of pairwise relatedness and inbreeding in the current population were weakly, but positively, correlated to molecular estimates. Thus, current studbooks are moderately effective at predicting genetic similarity when founder relatedness is known. Finally, we found that captive and wild populations did not differ in allelic frequencies, and conservation efforts to maintain diversity have been successful with no significant decrease in diversity over the last three generations.

**Supplementary Information:**

The online version contains supplementary material available at 10.1007/s10592-022-01429-7.

## Introduction

The need for conservation actions are rapidly increasing as biodiversity declines with the onset of the sixth mass extinction, which has already caused severe population declines in 30–50% of vertebrates worldwide (Ceballos et al. 2015, 2017, 2020). Current conservation strategies to minimize population declines and species extinction include in situ and ex situ methods. In situ strategies occur in a species’ native environment and can include food supplementation, translocation of individuals, and predator-free enclosures (Limoges et al. 2013; Kyle et al. 2017). In situ measures often provide short-term population increases with relatively minimal disturbance to the population. However, such measures are often slow to materialize and require relatively stable populations to be successful (Limoges et al. 2013; Kyle et al. 2017). In addition, severe population declines can occur before a species is recognized as endangered (Peters et al. 2015), at which point in situ approaches may be insufficient for conservation. Populations considered ‘critically endangered’, for example, may require more interventionistic approaches to avoid extinction (Peters et al. 2015).

Ex situ conservation generally consists of moving individuals out of their natural environments into artificial habitats, such as zoos or aquaria, with the intent of preventing imminent extinction (Canessa et al. 2015, Brichieri-Colombi et al*.* 2019). Populations facing extreme threats may be removed in their entirety or captive breeding programs may be implemented, in which case a subset of individuals are brought into a controlled environment and their offspring are released to reinforce the wild population (Canessa et al. 2015). Captive breeding programs have prevented the extinction of California condors (*Gymnogyps californianus*; Ralls and Ballou 2004), black-footed ferrets (*Mustela nigripes*; Wisely et al. 2003), and red wolves (*Canis lupus rufus*; Hedrick and Fredrickson 2008) among others (Barbanti et al. 2019). However, captive breeding programs are not always successful as they are constrained by certain logistical and financial issues. For example, some species fail to thrive and propagate in captive environments (Robin 2003). In species that will breed, limited capacity, limited population size, and accidental breeding of closely related individuals can lead to high levels of inbreeding (Kyle et al. 2017; Barbanti et al. 2019). Inbreeding is a major concern in conservation efforts as it can lead to reduced fitness (Ólafsdóttir and Krisjánsson 2008; Knief et al. 2015). Consequently, one of the foremost long-term challenges in captive breeding programs is preventing inbreeding despite a limited gene pool (Rollinson et al. 2014; Kyle et al. 2017).

Captive-breeding programs have attempted to address the concerns of inbreeding and loss of genetic diversity by using pedigree-based methods, traditionally using studbooks (records of past breeding events) to track ancestry and recommend future breeding pairs (Ivy et al. 2009; Lacy et al. 2012; Kyle et al. 2017). However, one concern with studbooks is the unknown relatedness amongst the initial breeding stock (founder population) or ‘new’ breeders later on. In the absence of prior information, these founders are often assumed to be unrelated, or relatedness may be inferred based on behavioural observations or geographic distance between source populations in the wild (Ivy et al. 2009; Witzenberger and Hochkirch 2011; Kyle et al. 2017). While estimates of relatedness and inbreeding among founder progeny become increasingly accurate within four generations (Huisman et al. 2016), the initial breeding events may result in unacceptable loss of diversity for populations already experiencing limited genetic variation (Frankham et al. 2017). Further, studbooks predict relatedness between pairs of individuals by assuming the law of averages from Mendelian inheritance across generations—half-siblings or grandparent-offspring dyads are expected to share 25% of their genes. In reality this will not be true for every pair of individuals as cross-over and meiotic reduction is random across gametes, meaning some dyads may share considerably more or less than 25% of their genes. This uncertainty can be exacerbated over generations leading to undesirable consequences in small populations. Nonetheless, mating pairs are typically selected based on high genetic dissimilarity according to studbook estimates, which can lead to differences between expected and true (realized) relatedness (Ivy et al. 2009; Witzenberger and Hochkirch 2011; Kyle et al. 2017).

**Fig. 1:**
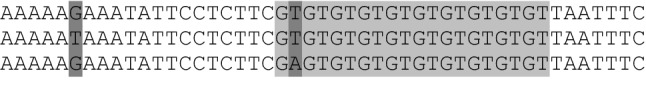
Three alleles of the same length displaying length homoplasy. Darkest highlighted regions indicate regions with sequence differences, light grey indicates microsatellite repeat sequence

The concerns associated with the use of studbooks have prompted the incorporation of molecular techniques to supplement, or even replace, traditional studbooks (Sekino et al. 2003; Ivy et al. 2016; Modesto et al. 2018). Unique genetic information is often acquired by genotyping individuals at microsatellite loci; markers that vary in the number of repeat units (length-based fragment analysis). This approach is relatively affordable and accessible, especially in non-model species with little molecular information available (Selkoe and Toonen 2006; Darby et al. 2016). However, this approach is prone to underestimating genetic diversity due to length homoplasy—alleles with identical length may not be identical by descent or by sequence (Fig. 1; Darby et al. 2016; Barbian et al. 2018). Consequently, length-based microsatellite genotyping typically detects only a subset of the genetic variation present. For species with very low genetic diversity, length-based fragment analysis may not reveal sufficient polymorphism to assist conservation and management (Darby et al. 2016; Barbian et al. 2018).

High-throughput amplicon sequencing (HTAS) (Darby et al. 2016; Barbian et al. 2018) is a targeted technique that sequences microsatellite loci, offering a bridge between traditional microsatellites and genome-wide SNP (single nucleotide polymorphism) methods. HTAS provides several benefits: (1) it overcomes length homoplasy by revealing SNPs, and other mutations, in addition to length variation; (2) it is cheaper than restriction-site associated SNP methods as microsatellite loci have typically already been identified for the species of interest or can be readily applied from closely related species; (3) future samples can easily be added because primers consistently amplify the same loci in separate runs overtime; and (4) much of the intial preparatory work can be done in a laboratory with limited equipment before being sent for sequencing, making this technique more accessible and further reducing costs. Importantly, HTAS has revealed 61–79% more diversity compared to traditional length-based fragment analysis in some species (Darby et al. 2016; Barbian et al. 2018).

In this study, we focus on the Vancouver Island marmot (*Marmota vancouverensis;* Swarth 1911; hereafter VIM) which is endemic to Vancouver Island and the only critically-endangered terrestrial mammal species in Canada (COSEWIC 2008, Roach 2017, Vancouver Island Marmot Recovery Team [VIMRT] 2017, COSEWIC 2019). The population declined in the early 1990s, likely as a result of increased predation and habitat modification through timber harvest, resulting in less than 30 wild individuals by 2003 (VIMRT 2017). Low numbers led to the initiation of an intensive captive breeding program in 1997 involving four different breeding facilities (Toronto Zoo, Calgary Zoo, Mountainview Conservation Centre, and Tony Barrett Mt Washington Marmot Recovery Centre). Between 2003 and 2020, 529 captive-bred offspring have been released back into the wild which has helped to recover the current wild population to ~ 200 individuals (Aaltonen et al. 2009, Brashares et al. 2010, Jackson et al. 2016, VIMRT 2017, Werner 2017, COSEWIC 2019, Lloyd et al. 2019).

In addition to behavioural considerations (Casimir et al. 2007), the captive breeding program uses a studbook to record breeding history and select new breeding pairs while attempting to minimize mean kinship (MK) and inbreeding coefficients following the recommendations of Ballou and Lacy (1995). Though our current pedigree contains a maximum of four generations, the inception of the VIM studbook was preceded by several years of behavioural observations in the wild which, along with geographically-dispersed populations, helped to estimate relatedness among most of the founders. The captive population has been self-sufficient since it’s establishment in 2001 until some wild individuals were supplemented in 2019 to boost the population size and the predicted genetic diversity.

Kruckenhauser et al. (2009) genotyped individuals from the wild VIM population during the decline in the mid to late 1990s; predating the release of captive-bred marmots into the wild (VIMRT 2017). Eleven polymorphic microsatellites showed low allelic richness (mean 2.1 alleles/locus) and three genetically-distinct population clusters (Kruckenhauser et al. 2009). The extant population is small, has likely undergone a genetic bottleneck due to severe decline, and is likely to display patterns of genetic drift that further decrease its genetic variation. Thus, we expected length-based fragment analysis to lack sufficient polymorphism to differentiate among individuals making relatedness and inbreeding inestimable. However, HTAS genotyping may uncover hidden variation and provide increased resolution to distinguish individuals. In this study, we used traditional length-based fragment analysis and HTAS genotyping of microsatellite loci in VIM to: (1) determine how much genetic diversity is hidden due to length homoplasy by comparing length-based genotypes to HTAS genotypes; (2) compare molecular estimates of pairwise relatedness and inbreeding with estimates derived from studbook ancestry; (3) compare the genetic diversity in the wild and captive populations; and (4) determine changes in allelic richness over time by comparing previous estimates to current length-based estimates.

## Materials and methods

### Sample origins and sequencing

**Fig. 2:**
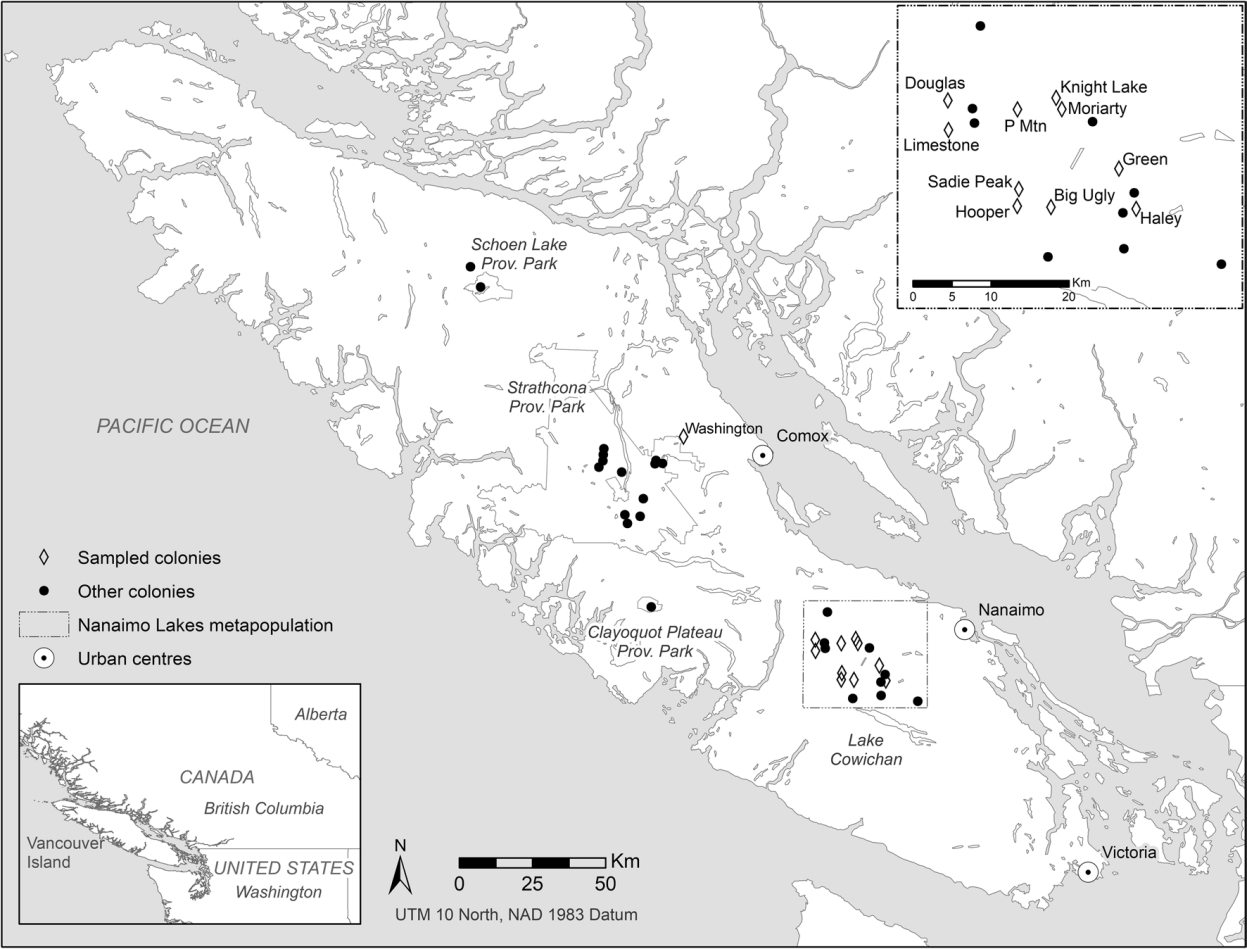
Map of Vancouver Island showing active marmot colonies. Colonies genotyped in this study are represented by white diamonds

Tissue and hair samples from captive and wild marmots were collected by the Calgary Zoo, the Toronto Zoo, and the Marmot Recovery Foundation. A total of 88 individuals sampled between 2005 and 2017 were selected based on their survival to the summer of 2018, and represented both wild and captive populations. Selected individuals included 51 captive-bred and 37 wild-born marmots. Wild marmots originated from ten colonies within the Nanaimo Lakes metapopulation (*n* = 33) and the Mt. Washington colony in the Strathcona metapopulation (*n* = 4) (Fig. 2). DNA was extracted from each tissue or hair sample (using a Qiagen DNA Blood and Tissue extraction kit) and amplified at 25 common Sciuridae microsatellite loci using QIAGEN TopTaq® Master Mix (Detailed PCR conditions and primers sequences for all loci are in Supplemental Table S1). Primers were redesigned for loci MA001, MA018 and 3b1 to reduce the length of the amplicon to less than 300 bp for sequencing. Illumina adapter overhang nucleotide sequences (Forward overhang: 5ʹ TCGTCGGCAGCGTCAGATGTGTATAAGAGACAG‐[locus specific sequence], Reverse overhang: 5ʹ GTCTCGTGGGCTCGGxAGATGTGTATAAGAGACAG‐[locus specific sequence]) were added to the 5’ ends of the locus-specific microsatellite primers to facilitate introduction of dual indices and Illumina sequencing adapters (Illumina 2013; Darby et al. 2016). Following amplification, the 25 PCR products were pooled at equal volumes for each individual. Pooled reactions were purified using Agencourt AMPure XP beads (Beckman) using a 1:1 ratio and used as template in a second PCR reaction. Indexing PCR reactions were performed in 25 μL total volume containing 2.5 μL of template, 12.5 μL of 2X KAPA HiFi HotStart Ready Mix (KAPA Biosystems), 2.5 μL of Nextera XT Index 1 primer (N7XX), 2.5 μL of Nextera Index 2 primer (S5XX); (Illumina), and 5 uL of PCR grade H_2_O. PCRs were cycled at 95 °C for 3 min followed by 8 cycles of 95 °C for 30 s, 55 °C for 30 s and 72 °C for 30 s and completed with a final extension at 72 °C for 5 min. Indexed microsatellite amplicons were pooled across individuals and again purified using Agencourt AMPure XP beads at a 1:1 ratio. The final library was sequenced on an Illumina MiSeq using a V3 2X300 bp sequencing kit and a 15% PhiX spike-in.

### Genotyping

In steps similar to Darby et al. (2016), raw sequence data were quality checked, trimmed and merged to produce text files containing both length and sequence information. Briefly, FastQC (Andrews 2010) was used to ensure the average sequence quality was > 20 and that sequences were not over-represented. Paired-end reads were trimmed for low quality bases (-trimns; -trimqualities) and overlapping reads were merged (-collapse) using AdapterRemoval v2 (Schubert et al. 2016). Merged reads were partitioned so that the number of unique reads per individual per primer pair could be determined using USEARCH (Edgar 2010) parameters -fastx_uniques, -fastaout, -sizeout and -tabbedout. These dereplicated reads were filtered by size and length to produce plain text files for each primer pair per individual. The ten read lengths with the highest frequency were used for genotyping. A detailed workflow of the pipeline has been placed on github (https://github.com/jazjanes/VI-marmots). Genotypes were scored manually by visualizing the typical microsatellite stutter profile using unique sequence length and the number of copies of each sequence per individual (Supplemental Figure S1). In determining individual genotypes, the minimum count required for a primary allele was 80 amplicons with a stutter peak within four base-pairs. For a genotype to be heterozygous the secondary allele needed a minimum of 20 amplicons with stutter peaks within four base-pairs, and the shortest allele needed to have the higher amplicon count of the two alleles. In the case of heterozygotes with two different sequences of the same length, the peak representing the second allele had to be greater than 80% of the first allele peak. Sequences with identical lengths were compared in BioEdit (Hall 1999) to identify hidden variation (Supplemental Figure S1). An additional data set was generated where the HTAS genotypes were simplified to create standard length-based genotypes where SNPs were ignored, different sequences of the same length were merged into single alleles, and some heterozygotes became homozygotes.

### Data analysis

Allelic richness, expected and observed heterozygosities were calculated using GenAlEx 6.5 (Peakall and Smouse 2006). We used GENEPOP (Raymond and Rousset 1995) to estimate linkage disequilibrium and deviations from Hardy–Weinberg equilibrium (HWE) with a Bonferroni correction to avoid Type 1 error (Rice 1989). Loci that deviated from HWE were not removed from subsequent analysis because we sampled across colonies and some colonies were represented by single individuals. Therefore, the violation of HWE is likely due to non-random sampling, which recent research has shown is not detrimental to subsequent analyses (Trevoy et al. 2018).

Independent Shapiro–Wilk tests revealed that three separate estimates of allelic richness (HTAS genotypes from all loci, length-based genotypes from all loci, and length-based genotypes from six loci in common with Kruckenhauser et al. (2009)) all deviated from a normal distribution. Hence, non-parametric Wilcoxon Signed-Rank tests were used to compare the allelic richness of HTAS vs. length-based genotypes, captive vs. wild individuals, and current length-based vs. previous length-based genotypes from Kruckenhauser et al. (2009). Individual observed heterozygosity (H_O_) was compared between captive vs. wild populations using a Mann–Whitney U test and between HTAS vs. length-based genotypes using both a Mann–Whitney and a Wilcoxon Signed-Rank test.

Molecular estimates of individual inbreeding coefficients (Ritland 1996) and pairwise relatedness were calculated using HTAS genotypes from all loci in COANCESTRY 2.0 (Wang 2011). A simulation with our data revealed that the TrioML estimator (Wang 2007) had the lowest variance and was thus used to calculate pairwise relatedness (Simulation results are in Supplemental Table S2). As inbreeding coefficients range from -1 to + 1 and TrioML relatedness coefficients range from 0 to 1, values outside of this range are artifacts of extreme homozygosity in the selected microsatellites (Wright 1922). We compared molecular inbreeding coefficients and estimates of pairwise relatedness between wild and captive-born individuals using Mann–Whitney U tests or Student’s t-tests where appropriate. Statistical tests were performed using the RealStats extension in Microsoft Excel (Zaiontz 2013) and all values were reported with mean ± SD.

Studbook estimates of inbreeding and pairwise kinship were calculated in the captive breeding management software PMx 1.6 (Lacy et al. 2012). Pairwise relatedness coefficients were calculated using R(xy) = 2*f(xy)/√{(1 + Fx)(1 + Fy)}, where f(xy) is the studbook kinship between individuals x and y, and Fx and Fy are the studbook inbreeding coefficients of individuals x and y (Crow and Kimura 1970; Galla et al. 2020). Due to non-normal distributions, we used nonparametric Spearman’s rank correlations to assess the relationships between molecular and studbook estimates of both individual inbreeding coefficients and pairwise relatedness. We also evaluated relatedness using a Pearson’s correlation and a Mantel test due to the presence of repeated values leading to rank ties and the dependency of pairwise matrices. All correlations included 47 captive-bred individuals with known parental ancestry going back at least two generations. Correlations were performed in R 4.0 (R Core Team 2020) and we used the *ape* 5.0 package (Paraidis and Schliep 2018) to complete the Mantel test with 999 permutations.

To investigate differences in allele frequencies among colonies and populations, we used the Bayesian clustering algorithm in STRUCTURE 2.3.4 (Pritchard et al. 2000). We analyzed wild and captive individuals simultaneously across 20 independent runs testing *K* = 1–5 using both HTAS genotypes from all loci, and length-based genotypes using only the six loci in common with Kruckenhauser et al (2009) in separate analyses. Runs included one million MCMC iterations preceded by 100,000 burn-in with correlated allele frequencies using an admixture model. We used StructureSelector (Li and Liu 2018) and CLUMPAK (Kopelman et al. 2015) to ensure our analyses converged, to determine the optimal number of clusters (considering only the mean Ln P(*K*) to avoid the recognized biased with Δ*K* (Janes et al. 2017; Cullingham et al. 2020), and to visualize cluster bar plots. In the event of *K* = 2, each cluster was reanalyzed separately to investigate the possibility of hidden substructure as recommended by Janes et al. (2017) using an individual *q*-value threshold of 0.9 for cluster membership.

## Results

After bioinformatic processing, we were left with an average of 9,581 ± 4,610 reads per individual, per locus across 25 loci and 88 individuals. Of the 25 loci examined, seven loci were eliminated as reads failed to reach genotyping thresholds in greater than 50% of individuals (2h10, 2h15, Bibl14, GS25, MA002, MS6, MS41), and another seven loci were removed because they were monomorphic (2g4, 3b1, Bibl18, Bibl31 GS12, MA066, MA091) leaving 11 loci for analysis (2g2, 2h4, 2h6, Bibl25, GS14, GS17, MA001, MA018, MS53, MS56, St10). Six of the 11 loci were in common with the loci used by Kruckenhauser et al. (2009; 2g2, GS17, MA018, MS53, MS56, St10). Six individuals were eliminated (one captive and five wild) because they failed to amplify in a minimum of 50% of 11 loci used for analysis, leaving 82 individuals remaining in the analysis; 50 captive-bred and 32 wild-born individuals from ten colonies in the Nanaimo Lakes metapopulation (*n* = 29) and Mt. Washington in the Strathcona metapopulation (*n* = 3). Of the 11 successful loci and 82 individuals genotyped, we sequenced an average of 9,854 ± 3,937 reads per individual, per locus, with all loci having a minimum of 3,508 average number of reads per individual. All loci were in linkage equilibrium and although eight of 11 loci deviated from Hardy–Weinberg equilibrium, they were retained in the analysis as specified in the methods (Table 1).

**Table 1:** Comparison of observed and expected heterozygosity and number of alleles between length-based and HTAS genotypes in the Vancouver Island marmot

	Length-based				HTAS				
Locus	A	H_O_	H_E_	n	A	H_O_	H_E_	n	Increase in alleles
2g2	3	0.28	0.47	78*	3	0.28	0.47	78*	
2h4	2	0.73	0.46	82*	3	0.73	0.47	82*	+ 1 (50%)
2h6	2	0.08	0.07	78*	2	0.08	0.07	78*	
Bibl25	2	0.16	0.35	74*	2	0.16	0.35	74*	
GS14	1	0.00	0.00	747	2	0.35	0.32	74*	+ 1 (100%)
GS17	2	0.31	0.49	77*	2	0.31	0.49	77*	
MA001	2	0.11	0.19	80*	2	0.11	0.19	80*	
MA018	4	0.51	0.69	77*	4	0.51	0.69	77*	
MS53	2	0.12	0.20	81*	2	0.12	0.20	81*	
MS56	2	0.34	0.28	82*	2	0.34	0.28	82*	
St10	2	0.05	0.48	80*	2	0.05	0.48	80*	
Mean	2.2	0.24	0.33	78.5	2.4	0.28	0.36	78.5	

Asterisks denote loci deviating from Hardy–Weinberg equilibrium

*H*_*O*_ observed heterozygosity, *H*_*E*_ expected heterozygosity, *A* alleles per locus, *n* sample size

Allelic richness from length-based and HTAS genotypes across 11 polymorphic loci revealed one to four alleles per locus (Table 1) with two novel alleles discovered using HTAS genotyping (8.3% increase in allelic richness). One of these new alleles was at a locus (GS14) that would otherwise have appeared to be monomorphic. These two new alleles decreased the probability of identity (two randomly selected individuals having the same genotype) from less than 1 in 3,000 (length-based) to less than 1 in 6,000 (HTAS). However, using sequence data to overcome length homoplasy did not significantly increase mean allelic richness across all 11 loci (length-based 2.2 ± 0.2, HTAS 2.4 ± 0.2; z = 1.4, n = 11, *P(one-tail)* = 0.08, Table 1). Interestingly, mean individual heterozygosity derived from HTAS genotypes (0.28 ± 0.14) was greater than length-based genotypes (0.25 ± 0.13) using a paired Wilcoxon test (z = 4.5, n = 82, *P* < 0.001) but this difference was not supported by an independent Mann–Whitney test (U = 2,924, n_1_ = n_2_ = 82, *P* = 0.08).

**Fig. 3:**
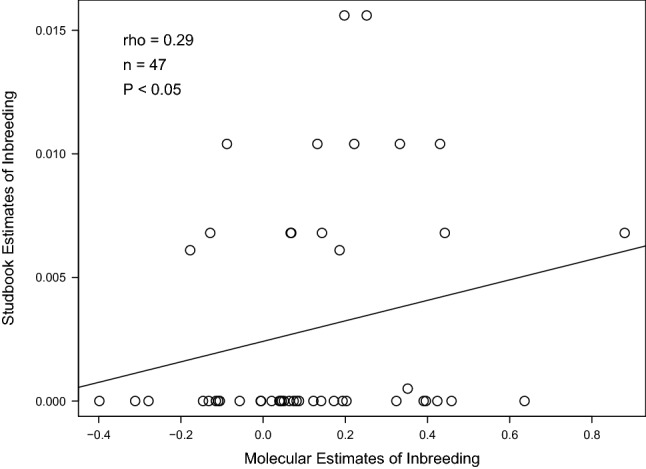
Correlation of studbook estimates and molecular estimates of inbreeding coefficients in Vancouver Island marmot (n = 47 individuals)

**Fig. 4:**
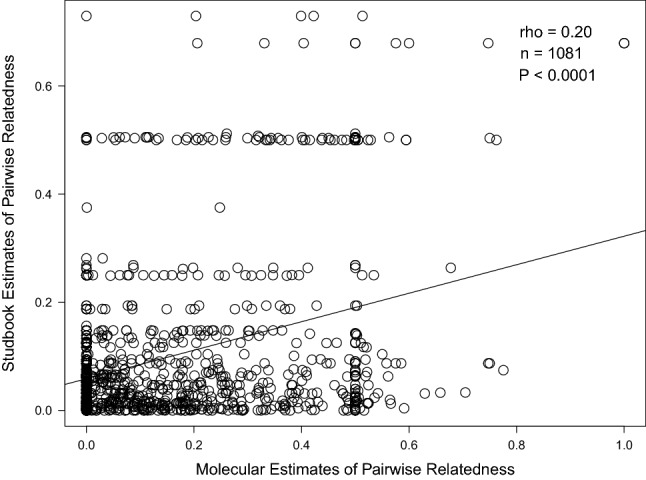
Correlation of studbook estimates and molecular estimates of pairwise relatedness in Vancouver Island marmot (n = 1081 pairwise comparisons among 47 individuals)

Molecular estimates of inbreeding (0.119 ± 0.247) were poorly, but positively, correlated with studbook estimates (0.003 ± 0.005; Spearman’s rho = 0.29, n = 47, *P* < 0.05, Fig. 3) with three individuals excluded from the analysis (two individuals had less than two generations worth of studbook information available and one had a molecular inbreeding value outside the theoretical range). Molecular estimates of pairwise relatedness (0.15 ± 0.19) were also weakly, but positively, correlated with studbook estimates (0.10 ± 0.14; Spearman’s rho = 0.20, n = 1081, *P* < 0.0001, Fig. 4). This relationship remained significant with a Pearson correlation (r = 0.34, *P* < 0.0001) and a Mantel test (z-stat = 25.6, *P* = 0.001).

Mean allelic richness of the captive population (2.3 ± 0.6) was not statistically different than the wild population across all 11 loci (2.4 ± 0.7; z = 1.0, *P* = 0.32), and we did not discover any private alleles among wild colonies (Table 2). Likewise, mean individual heterozygosity did not vary between the captive (0.29 ± 0.14) and wild (0.25 ± 0.14) populations (U = 681.5, n_1_ = 50, n_2_ = 32, *P* = 0.13). However, molecular inbreeding coefficients were greater in the wild (0.41 ± 0.14) than the captive (0.15 ± 0.04) population (U = 579.5, n_1_ = 50, n_2_ = 32, *P* < 0.04), although this was partially driven by three outliers (2 wild, 1 captive) with values outside of the theoretical limits of the estimate (− 1 to + 1). With these three outliers removed inbreeding coefficients became normally distributed and the wild population was only marginally greater than the captive population (0.25 ± 0.05, 0.13 ± 0.04 respectively; t(77) = 1.99, *P* = 0.051). Pairwise relatedness estimates among wild marmots (0.14 ± 0.18) were not different from the captive (0.15 ± 0.19) population (U = 312,807.5, n_1_ = 1,081, n_2_ = 595, *P* = 0.17).

**Table 2:** Comparison of HTAS and length-based genotypes from this study and the previous study by Kruckenhauser et al. (2009), including allelic richness (*A*), number of private alleles (*pA*), observed (*H*_*O*_) and expected heterozygosity (*H*_*E*_), and number of Vancouver Island marmots genotyped (*n*)

	HTAS genotypes (this study)					Length-based genotypes (this study)					Length-based genotypes (Kruckenhauser et al. 2009)				
	*A*	*pA*	*H*_*O*_	*H*_*E*_	*n*	*A*	*pA*	*H*_*O*_	*H*_*E*_	*n*	*A*	*pA*	*H*_*O*_	*H*_*E*_	*n*
*Populations*															
Captive	2.36	0	0.30	0.36	50	2.18	0	0.26	0.33	50					
Mt. Washington	1.73	0	0.35	0.30	3	1.64	0	0.29	0.26	3	1.27	5	0.07	0.08	11
Nanaimo Lakes	2.36	0	0.24	0.36	29	2.18	0	0.22	0.34	29	1.40	6	0.21	0.2	94
*Nanaimo Lakes colonies*															
Green	1.73	0	0.32	0.33	2	1.73	0	0.32	0.33	2	1.55	0	0.29	0.23	9
Haley	2.09	0	0.33	0.36	7	2.00	0	0.28	0.31	7	1.64	0	0.18	0.18	10
Ugly	1.27	0	0.36	0.18	1	1.27	0	0.36	0.18	1	1.36	0	0.13	0.12	5
Douglas	1.00	0	0.18	0.09	1	1.00	0	0.18	0.09	1					
Hooper	1.64	0	0.21	0.24	5	1.64	0	0.21	0.24	5					
Knight_Lake	0.73	0	0.00	0.00	3	0.73	0	0.00	0.00	3					
Limestone	1.27	0	0.27	0.14	1	1.27	0	0.27	0.14	1					
Moriarty	1.82	0	0.21	0.27	6	1.73	0	0.19	0.26	6					
P_Mtn	0.91	0	0.00	0.00	1	0.91	0	0.00	0.00	1					
Sadie	1.46	0	0.32	0.22	2	1.36	0	0.23	0.17	2					
K44A											1.55	0	0.29	0.23	27
Pat Lake											1.45	0	0.24	0.19	12
Sherk Lake											1.36	0	0.25	0.18	4
Mt. Franklin											1.55	0	0.15	0.15	21

**Fig. 5:**
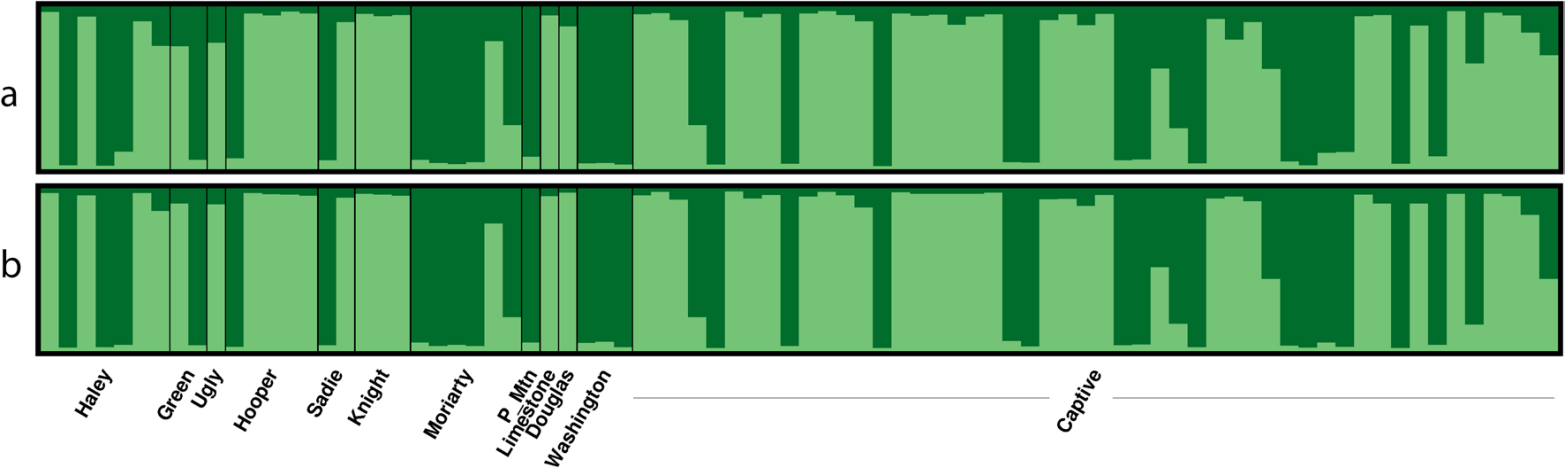
Individual membership likelihoods across two genetic clusters in wild and captive Vancouver Island marmots (n = 82) using (**a**) sequence-based genotypes from 11 microsatellite loci, and (**b**) length-based genotypes from six microsatellite loci

A Bayesian STRUCTURE analysis using the HTAS genotypes from all 11 loci identified two genetic clusters (*K* = 2) across all runs (Supplemental Figures S2-S3). However, there was no obvious geographic pattern of cluster separation between the wild and captive populations or across wild colonies as both clusters were represented equally in wild and captive populations (Fig. 5a). A second STRUCTURE analysis using only length-based genotypes from the six loci in common with Kruckenhauser et al. (2009) did not change the number of clusters, or the representation of genetic structure across populations or colonies (Fig. 5b, Supplemental Figures S2-S4). Furthermore, subsequent STRUCTURE analyses within each cluster did not reveal any hidden substructure using either the full HTAS dataset or the reduced dataset with only six loci.

Using only the six loci in common with Kruckenhauser et al. (2009), allelic richness from length-based genotypes was 2.5 ± 0.8 which was not statistically different from 2.2 ± 0.4 estimated by Kruckenhauser et al. (2009) (z = 1.4, *P* = 0.16). Expanding our dataset to ten loci, including the same six polymorphic loci in common between the two studies plus Bibl18 (monomorphic in our study but polymorphic in Kruckenhauser et al. 2009) and three other loci (Bibl31, GS12, MA091) deemed monomorphic in both studies, still did not reveal a significant change in mean allelic richness over time (z = 0.6, *P* = 0.56).

## Discussion

We compared the genetic diversity of VIM using both length-based microsatellite genotyping and next-generation sequencing to assess the ability of the HTAS approach to detect greater genetic variation in a species with low genetic diversity. Additionally, we used HTAS to compare molecular and studbook-derived estimates of relatedness and inbreeding. We determined that the HTAS approach did not detect significantly more genetic diversity compared to traditional fragment length analysis. However, the field behavioural observations used to establish the studbook created a reliable and effective way of minimizing inbreeding. Wild and captive populations were similar in all aspects except that the wild population had marginally higher molecular estimates of inbreeding. Genetic variation in VIM appears to have remained constant since the previous estimates, although some population structure may have degraded.

### Length-based vs HTAS genotyping

Despite our expectations, the HTAS approach did not lead to a significant increase in allelic richness compared to traditional length-based genotyping. Only two additional alleles were identified across 11 loci. In contrast, chimpanzees (*Pan troglodytes*) had a 61% increase in mean allelic richness using HTAS (Barbian et al. 2018) while muskrats (*Ondatra zibethicus*) saw a 79% increase (Darby et al. 2016). However, the conflicting results may be attributable to the relatively low genetic diversity of VIM. For example, the mean allelic richness using length-based genotyping was 6.4 in chimpanzee (Barbian et al. 2018) and 14.9 in muskrat (Darby et al. 2016), while VIM was just 2.1 historically (Kruckenhauser et al. 2009) and 2.2 in this study. Likewise, mean expected heterozygosity was 0.75 in chimpanzee, 0.82 in muskrat but only 0.33 in our study. VIM is an endemic species with historically small population sizes; it is likely to be heavily impacted by genetic drift which would contribute to extremely limited genetic diversity. The Vancouver lamprey (*Entosphenus macrostomus*), the only other species endemic to Vancouver Island that we are aware of (although we recognize there are several endemic subspecies), also exhibits low genetic variation (mean allelic richness of 3.1, mean expected heterozygosity of 0.50; Taylor et al. 2012). Both studies by Darby et al. (2016) and Barbian et al. (2018) showed that the HTAS approach increased H_O_ at all loci, while we only saw an increase in two of the eleven loci. However this was enough to significantly increase mean individual H_O_ when using a more sensitive paired Wilcoxon test, though the lack of significance with the Mann–Whitney test suggests this difference was small and not robust. This potential increase is the result of finding heterozygotes with the HTAS approach that would have otherwise appeared to be homozygotes using traditional length-based genotyping (two different sequences of the same length). In all, despite the reduced cost and increased accessibility of the HTAS approach, this technique may have limited applications in the conservation and management of endangered species with reduced diversity as there are also fewer alleles hidden by homoplasy.

### Studbook vs HTAS estimates

We found that molecular and studbook estimates of both inbreeding and pairwise relatedness were positively correlated, although both relationships contained considerable unexplained variance. Generally, molecular genotypes tend to overestimate relatedness due to the presence of identical-by-state (but not identical-by-descent) alleles which artificially increase similarity among individuals (Taylor 2015; Taylor et al. 2015). In contrast, studbooks can underestimate relatedness by not accounting for relations prior to the start of record-keeping (Wells et al. 2018; Hogg et al. 2019). Also, studbook estimates are based on the 50% rule of Mendelian inheritance which can under or overestimate true relatedness between kin (Galla et al. 2020).

Given the low allelic richness in VIM, our molecular data are challenged to calculate relatedness and inbreeding with a high degree of confidence, which likely contributes in part to the unexplained variance (Taylor 2015; Taylor et al. 2015; Galla et al. 2020). However, our studbook greatly benefitted from prior behavioural observations to help estimate relatedness among founders. This approach is highly recommended as it can improve the reliability of kinship and inbreeding estimates, and minimize unknown relationships among founders (Russell et al. 1994, Kennedy et al. 2014, Taylor 2015, Frankham et al. 2017, Hogg et al. 2019, Galla et al. 2021). In this sense, our studbook is likely more accurate in estimating relatedness compared to studies that did not have any prior information during inception. However, new wild marmots have recently been recruited into the captive breeding program but are lacking pedigree data to estimate how they might be related to captive individuals. This presents a drawback to relying on the studbook alone, suggesting a hybrid approach of both behavioural and molecular data sets is likely ideal (Galla et al. 2020, Galla et al. 2021).

### Wild vs captive VIM

The loss of genetic diversity as a result of captive breeding is a known concern, one that is often followed by the recommendation that molecular markers be incorporated into studbook data (Ivy et al. 2009; Hogg et al. 2019; Ayala-Burbano et al. 2020). The VIM captive population was originally established with individuals from as many colonies as possible and hence we might expect to see limited differences between the wild and captive populations. Indeed, our results confirmed our expectations as we found no difference in allelic richness or individual heterozygosity between populations. Furthermore, our STRUCTURE analyses demonstrated that allele frequencies did not vary between the wild and captive populations. The maintenance of VIM genetic diversity speaks to the success of capturing genetic variation in the wild, incorporating behavioural data early on, and the diligent efforts to maintain diversity through the selection of breeding pairs (VIMRT 2017). Interestingly, our results suggested that the captive population was possibly less inbred than the wild and there may be multiple reasons for this. First, the statistical significance was mostly driven by three data points (these individuals had inbreeding values greater than one, which is outside of theoretical limits) which once removed resulted in only a marginally significant difference. Second, the breeding program intentionally minimized inbreeding in the captive population, while wild marmot colonies are usually geographically separated, small populations with limited gene flow (VIMRT 2017; COSEWIC 2019). Third, the accuracy of inbreeding and relatedness estimates is known to be dramatically reduced when using few low diversity microsatellites (Taylor et al. 2015). Genotyping techniques with greater resolution would provide more confidence around these estimates therefore, we remain conservative about our ability to accurately quantify inbreeding coefficients and what these values imply about the two populations as a whole. Consequently, while the captive population may be less inbred than the wild population, this does not indicate that the wild individuals are severely inbred.

### Genetic diversity in the wild over time.

Due to the severe population decline since the previous study by Kruckenhauser et al. (2009, VIMRT 2017, COSEWIC 2019), we anticipated a noticeable decrease in genetic diversity in the wild. Contrary to expectations, our estimate of mean allelic richness in VIM was not significantly different from the previous estimate (Kruckenhauser et al. 2009). In fact, we identified two new length-based alleles in our study (at loci 2g2 and MA018) that were not identified in the previous study. The most likely reason for this discrepancy is incomplete sampling of the population as these new alleles were relatively low in frequency in our study. While the mutation of new alleles between sampling events (approximately three generations; VIMRT 2017) seems unlikely, this remains a possibility.

Overall, we failed to observe a decrease in genetic variation as would be expected through drift. This may be attributed to the pre-existing low diversity as well as successful conservation management. Conservation efforts have been focused on maintaining gene flow between colonies through translocations and releases, and careful management and implementation of the captive-breeding program by the Marmot Recovery Foundation and the Calgary and Toronto Zoos. In fact, the software *PMx* estimates that our pedigree has retained 96% of the original genetic diversity from the founding captive population. However, translocations among colonies and releases from captive populations are not without consequence; population genetic structure seems to have eroded since the initial assessment before translocations and releases began. Kruckenhauser et al. (2009) identified three genetic clusters (*K* = 3), including one cluster for Mt. Washington and two clusters split across the Nanaimo Lakes metapopulation. Our study also shows two clusters split across the Nanaimo Lakes metapopulation but fails to separate Mt. Washington as a unique cluster. Although only three samples from Mt. Washington were genotyped in the current study, these individuals did not have any private alleles which is in contrast to Kruckenhauser et al. (2009) who found private alleles at five loci. Additionally, one locus (Bibl18), identified by Kruckenhauser et al. (2009) as containing a fixed but private allele at Mt. Washington, now appears to be monomorphic across all individuals sampled. While our results may be due to small sample size, they are more likely suggesting that the pre-existing genetic structure between Mt. Washington (Strathcona metapopulation) and the Nanaimo Lakes metapopulation has been eroded. This is not surprising considering the Marmot Recovery Team determined the genetic variance in the Mt. Washington colony would be best intermixed across all populations, leading to extensive translocations among wild colonies and releases of marmots to and from the Mt. Washington colony (VIMRT 2017; Lloyd et al. 2019).

## Conclusions

We determined that genetic diversity of the Vancouver Island marmot appears to be unaffected by several generations of captive breeding, with wild and captive populations maintaining allelic diversity over time. This is likely attributed to the early implementation of behavioural and molecular data in the establishment of the studbook and diligent efforts to minimize inbreeding among mating pairs ever since. Moving forward, we recommend current and future captive breeding programs incorporate molecular data to help establish or improve studbooks and inform mate selection. Conversely to our expectations, we also determined that while HTAS genotyping did reveal a few hidden alleles, these did not lead to a significant increase in estimates of genetic diversity, indicating this technique may have limited value in species with especially low genetic variation. The HTAS technique should still be explored further for its potential suitability in the conservation of endangered species, although some species, like the Vancouver Island marmot, may benefit more by using a sequencing technique that has the potential to capture a greater number of genome wide markers (e.g. restriction-site associated DNA sequencing or whole genome sequencing).

## Supplementary Information

Below is the link to the electronic supplementary material.

Supplementary file1 (DOCX 548 kb)

Supplementary file2 (XLSX 81 kb)

## Data Availability

All data generated or analysed during this study are included in this published article (and its supplementary information files).

## Code availability

The script to organize sequence data is available at https://github.com/jazjanes/VI-marmots.
