# Supplementary material for: Genetic management on the brink of extinction: sequencing microsatellites does not improve estimates of inbreeding in wild and captive Vancouver Island marmots (Marmota vancouverensis)
Source: Conserv Genet. 2022 Jan 16;23(2):417–28. doi: 10.1007/s10592-022-01429-7 (PMC8948115; doi:10.1007/s10592-022-01429-7)
Supplement: Supplementary file 1 — Supplementary file1 (DOCX 548 kb) [file 10592_2022_1429_MOESM1_ESM.docx]

**Supplementary Information**

Genetic management on the brink of extinction: sequencing microsatellites does not improve estimates of inbreeding in wild and captive Vancouver Island marmots (*Marmota vancouverensis*)

Kimberley G. Barrett^1*^ (0000-0002-5191-5900), Geneviève Amaral^1§^, Melanie Elphinstone^1^, Malcolm L. McAdie^2^, Corey S. Davis^3^ (0000-0003-4362-0659), Jasmine K Janes^1,4^ (0000-0002-4511-2087), John Carnio^2^, Axel Moehrenschlager^5^ (0000-0003-2789-0376), Jamieson C. Gorrell^1,6^ (0000-0001-8235-2461)

1. Biology Department, Vancouver Island University, Nanaimo, BC, Canada, V9R 5S5

2. Marmot Recovery Foundation, Nanaimo, BC, Canada, V9R 6X9

3. Department of Biological Sciences, University of Alberta, Edmonton, AB, Canada, T6G 2R3

4. School of Environmental and Rural Science, University of New England, Armidale, NSW, Australia, 2351

5. Centre for Conservation Research, Calgary Zoological Society, Calgary, AB, Canada, T2E 7V6

6. Corresponding author: Jamie.Gorrell@viu.ca

*Present address: Department of Biological Sciences, University of Alberta, Edmonton, AB, Canada, T6G 2R3

^§^Present address: Island Medical Program, University of Victoria, 3800 Finnerty Road, Victoria, BC, Canada, V8P 5C2

**Table S1** PCR primers and conditions. F = forward, R = reverse, Alleles = Number of alleles detected; length of sequence (letters denote sequence variants of the same length). Asterisks (*) indicate which primers were redesigned in this study to reduce the length of the amplicon.

| **Locus** | **Reference/ GenBank Accession #** | **Primers** | **PCR conditions** | **Master mix** | **Alleles** |
| --- | --- | --- | --- | --- | --- |
| 2h4 | GQ294553 | F:TGTAGGTGTTGATGTTGAATGA  R:TCCCTGCCACAAGAAATA | 94ºC 5 min, (94ºC 20s, 58ºC 20s, 70ºC 20s) x2, (94ºC 20s, 54ºC 20s, 70ºC 20s) x36, 72ºC 2 min | 5μL Top Taq, 1μL of 10μM Primer, 2μL H_2_O, 2μL DNA | 3; 153, 159A, 159B |
| 2h6 | Kyle *et al,* 2004 | F:TAACTACACATTTGACTTTCTGC  R:ATCCCCAGCACCACATAC | 94ºC 5 min, (94ºC 20s, 58ºC 20s, 70ºC 20s) x2, (94ºC 20s, 56ºC 20s, 70ºC 20s) x3, (94ºC 20s, 54ºC 20s, 70ºC 20s) x31, 72ºC 2 min | 5μL Top Taq, 1μL of 10μM Primer, 1μL H_2_O, 3μL DNA | 2; 188,190 |
| 2h10 | Kyle *et al,* 2004 | F:GAATCCGAGTTGCCCAGTCC  R:CAGACAGGGACACGCACACG | 94ºC 5 min, (94ºC 20s, 58ºC 20s, 70ºC 20s) x2, (94ºC 20s, 56ºC 20s, 70ºC 20s) x3, (94ºC 20s, 54ºC 20s, 70ºC 20s) x31, 72ºC 2 min | 5μL Top Taq, 1μL of 10μM Primer, 1.7μL H_2_O, 0.3μL Mg^2+^, 2μL DNA | FAILED |
| 2h15 | Kyle *et al,* 2004 | F:TGGTTACGAAGATGGGAGAC  R:CTGGGCTGGAAGAACTGG | 94ºC 5 min, (94ºC 20s, 58ºC 20s, 70ºC 20s) x2, (94ºC 20s, 56ºC 20s, 70ºC 20s) x3, (94ºC 20s, 54ºC 20s, 70ºC 20s) x31, 72ºC 2 min | 5μL Top Taq, 1μL of 10μM Primer, 1.7μL H_2_O, 0.3μL Mg^2+^, 2μL DNA | FAILED |
| 2g2 | Kyle *et al,* 2004 | F:TGAACTGGGTCTTGAGGTCT  R:GTCTGCTCTGCTCTCCATCA | 94ºC 5 min, (94ºC 20s, 60ºC 20s, 70ºC 20s) x2, (94ºC 20s, 50ºC 20s, 70ºC 20s) x36, 72ºC 2 min | 5μL Top Taq, 1μL of 10μM Primer, 2μL H_2_O, 2μL DNA | 3; 120, 124, 126 |
| 2g4 | Kyle *et al,* 2004 | F:TAAGGCTGAATAATATTCCTCT  R:CAAACAACCCGAGTAGACAT | 94ºC 5 min, (94ºC 20s, 60ºC 20s, 70ºC 20s) x2, (94ºC 20s, 50ºC 20s, 70ºC 20s) x36, 72ºC 2 min | 5μL Top Taq, 1μL of 10μM Primer, 2μL H_2_O, 2μL DNA | 1; 140 |
| 3b1 | Kyle *et al,* 2004 | *F:CAAATGCACAGGTAATGCTG  *R:TTCAGGGAACTGATTTTGGA | 94ºC 5 min, (94ºC 20s, 62ºC 20s, 70ºC 20s) x2, (94ºC 20s, 60ºC 20s, 70ºC 20s) x2, (94ºC 20s, 58ºC 20s, 70ºC 20s) x10, (94ºC 20s, 56ºC 20s, 70ºC 20s) x10, (94ºC 20s, 54ºC 20s, 70ºC 20s) x10, 72ºC 2 min | 5μL Top Taq, 1μL of 10μM Primer, 1.7μL H_2_O, 0.3μL Mg^2+^, 2μL DNA | 1; 195 |
| Bibl4 | Klinkicht, 1993; Goossens *et al*, 1998 | F:CCTAGGTTCAGTCTTCAAC  R:TGGTGTTGCCATTGTTCTG | 94ºC 5 min, (94ºC 20s, 56ºC 20s, 70ºC 20s) x2, (94ºC 20s, 50ºC 20s, 70ºC 20s) x34, 72ºC 2 min | 5μL Top Taq, 1μL of 10μM Primer, 2μL H_2_O, 2μL DNA | FAILED |
| **Locus** | **Reference/ GenBank Accession #** | **Primers** | **PCR conditions** | **Master mix** | **Alleles** |
| Bibl18 | Klinkicht, 1993; Goossens *et al*, 1998 | F:ATGGTCATGGAAGGGAAGG  R:GCATCTTCACAGTTGATCT | 94ºC 5 min, (94ºC 20s, 59ºC 20s, 70ºC 20s) x2, (94ºC 20s, 53ºC 20s, 70ºC 20s) x33, 72ºC 2 min | 5μL Top Taq, 1μL of 10μM Primer, 2μL H_2_O, 2μL DNA | 1; 138 |
| Bibl25 | Klinkicht, 1993; Goossens *et al*, 1998 | F:CTCATGACTATGGCAGCC  R:AGAACCTTGATTTAGCAGTAG | 94ºC 5 min, (94ºC 20s, 62ºC 20s, 70ºC 20s) x2, (94ºC 20s, 55ºC 20s, 70ºC 20s) x36, 72ºC 2 min | 5μL Top Taq, 1μL of 10μM Primer, 2μL H_2_O, 2μL DNA | 2; 137, 141 |
| Bibl31 | Klinkicht, 1993; Goossens *et al*, 1998 | F:TTACACCTTCTCTGGCTCC  R:TCTGAGCGGATTGTCTTTAT | 94ºC 5 min, (94ºC 20s, 62ºC 20s, 70ºC 20s) x2, (94ºC 20s, 55ºC 20s, 70ºC 20s) x36, 72ºC 2 min | 5μL Top Taq, 1μL of 10μM Primer, 2μL H_2_O, 2μL DNA | 1; 159 |
| GS12 | Stevens *et al*, 1997 | F:CCAAGAGAGGCAGTCGTCCAG  R:TCAGAGCAGAGCACTTACAGA | 94ºC 5 min, (94ºC 20s, 58ºC 20s, 70ºC 20s) x2, (94ºC 20s, 54ºC 20s, 70ºC 20s) x36, 72ºC 2 min | 5μL Top Taq, 1μL of 10μM Primer, 2μL H_2_O, 2μL DNA | 1; 143 |
| GS14 | Stevens *et al*, 1997 | F:CAGGTGGGTCCATAGTGTTAC  R:TTGTGCCTCAGCATCTCTTTC | 94ºC 5 min, (94ºC 20s, 58ºC 20s, 70ºC 20s) x2, (94ºC 20s, 54ºC 20s, 70ºC 20s) x36, 72ºC 2 min | 5μL Top Taq, 1μL of 10μM Primer, 2μL H_2_O, 2μL DNA | 2; 242A, 242B |
| GS17 | Stevens *et al*, 1997 | F:CAATTCGTGGTGGTTATATC  R:CTGTCAACCTATATGAACACA | 94ºC 5 min, (94ºC 20s, 50ºC 20s, 70ºC 20s) x2, (94ºC 20s, 48ºC 20s, 70ºC 20s) x2, (94ºC 20s, 46ºC 20s, 70ºC 20s) x2, (94ºC 20s, 44ºC 20s, 70ºC 20s) x2, (94ºC 20s, 42ºC 20s, 70ºC 20s) x2, (94ºC 20s, 40ºC 20s, 70ºC 20s) x29, 72ºC 2 min | 5μL Top Taq, 1μL of 10μM Primer, 1.2μL H_2_O, 0.8μL Mg^2+^, 2μL DNA | 2; 150, 154 |
| GS25 | Stevens *et al*, 1997 | F:CCAGCATGGGGGAGAGAGAG  R:CTTGTCATTTATCCATTCATAG | 94ºC 5 min, (94ºC 20s, 56ºC 20s, 70ºC 20s) x2, (94ºC 20s, 50ºC 20s, 70ºC 20s) x34, 72ºC 2 min | 5μL Top Taq, 1μL of 10μM Primer, 2μL H_2_O, 2μL DNA | FAILED |
| MA001 | da Silva *et al*, 2003 | F:AGGGGAACAGAACCAAAAGG  *R:TCAGGAAAGAAATCAGACAAATC | 94ºC 5 min, (94ºC 20s, 62ºC 20s, 70ºC 20s) x2, (94ºC 20s, 60ºC 20s, 70ºC 20s) x2, (94ºC 20s, 58ºC 20s, 70ºC 20s) x10, (94ºC 20s, 56ºC 20s, 70ºC 20s) x10, (94ºC 20s, 54ºC 20s, 70ºC 20s) x10, 72ºC 2 min | 5μL Top Taq, 1μL of 10μM Primer, 1.7μL H_2_O, 0.3μL Mg^2+^, 2μL DNA | 2; 151, 153 |
| MA002 | da Silva *et al*, 2003 | F:CATTTAGACGCACATTTTG  R:GGGATGGAGAATGAGGAAG | 94ºC 5 min, (94ºC 20s, 64ºC 20s, 70ºC 20s) x2, (94ºC 20s, 62ºC 20s, 70ºC 20s) x2, (94ºC 20s, 60ºC 20s, 70ºC 20s) x2, (94ºC 20s, 58ºC 20s, 70ºC 20s) x2, (94ºC 20s, 56ºC 20s, 70ºC 20s) x2, (94ºC 20s, 55ºC 20s, 70ºC 20s) x25, 72ºC 2 min | 5μL Top Taq, 1μL of 10μM Primer, 1.7μL H_2_O, 0.3μL Mg^2+^, 2μL DNA | FAILED |
| **Locus** | **Reference/ GenBank Accession #** | **Primers** | **PCR conditions** | **Master mix** | **Alleles** |
| MA018 | da Silva *et al*, 2003 | *F:TGTCTTGCCTATTCTTGGTTCT  *R:AGCTTTCCCCAAACTGGTAT | 94ºC 5 min, (94ºC 20s, 62ºC 20s, 70ºC 20s) x2, (94ºC 20s, 60ºC 20s, 70ºC 20s) x3, (94ºC 20s, 58ºC 20s, 70ºC 20s) x31, 72ºC 2 min | 5μL Top Taq, 1μL of 10μM Primer, 2μL H_2_O, 2μL DNA | 4; 157, 163, 167,169 |
| MA066 | da Silva *et al*, 2003 | F:AATATGTTAAGGCAGTTCTAGC  R:GTTTCTTCCTGATATGGAAAGATGATGT | 94ºC 5 min, (94ºC 20s, 62ºC 20s, 70ºC 20s) x2, (94ºC 20s, 55ºC 20s, 70ºC 20s) x36, 72ºC 2 min | 5μL Top Taq, 1μL of 10μM Primer, 2μL H_2_O, 2μL DNA | 1; 224 |
| MA091 | da Silva *et al*, 2003 | F:CCTGTGTGAGTCCTGGAGTC  R:AGCCATTTAGGTTACATCTGC | 94ºC 5 min, (94ºC 20s, 64ºC 20s, 70ºC 20s) x2, (94ºC 20s, 62ºC 20s, 70ºC 20s) x2, (94ºC 20s, 60ºC 20s, 70ºC 20s) x2, (94ºC 20s, 58ºC 20s, 70ºC 20s) x2, (94ºC 20s, 56ºC 20s, 70ºC 20s) x2, (94ºC 20s, 55ºC 20s, 70ºC 20s) x25, 72ºC 2 min | 5μL Top Taq, 1μL of 10μM Primer, 1.7μL H_2_O, 0.3μL Mg^2+^, 2μL DNA | 1; 165 |
| MS41 | Hanslick and Kruckenhauser, 2000 | F:GGTGTATATGGGAATAGGGGG  R:GCCTTCAAATCAAAGCAGGTTG | 94ºC 5 min, (94ºC 20s, 59ºC 20s, 70ºC 20s) x2, (94ºC 20s, 53ºC 20s, 70ºC 20s) x33, 72ºC 2 min | 5μL Top Taq, 1μL of 10μM Primer, 2μL H_2_O, 2μL DNA | FAILED |
| MS53 | Hanslick and Kruckenhauser, 2000 | F:ATTGAGGAGCAGCATCTAGG  R:TCAGGGAAAGGCAGACCTG | 94ºC 5 min, (94ºC 20s, 59ºC 20s, 70ºC 20s) x2, (94ºC 20s, 53ºC 20s, 70ºC 20s) x33, 72ºC 2 min | 5μL Top Taq, 1μL of 10μM Primer, 2μL H_2_O, 2μL DNA | 2; 146, 148 |
| MS56 | Hanslick and Kruckenhauser, 2000 | F:CAGACTCCCACCAGTGAC  R:CCTGATCTATGTAGGTTCCAT | 94ºC 5 min, (94ºC 20s, 59ºC 20s, 70ºC 20s) x2, (94ºC 20s, 53ºC 20s, 70ºC 20s) x33, 72ºC 2 min | 5μL Top Taq, 1μL of 10μM Primer, 2μL H_2_O, 2μL DNA | 2; 102, 104 |
| MS6 | Hanslick and Kruckenhauser, 2000 | F:CTGATGGGGTTAAGATTGCC  R:CCCCACTGACCCACCTCC | 94ºC 5 min, (94ºC 20s, 59ºC 20s, 70ºC 20s) x2, (94ºC 20s, 53ºC 20s, 70ºC 20s) x33, 72ºC 2 min | 5μL Top Taq, 1μL of 10μM Primer, 2μL H_2_O, 2μL DNA | FAILED |
| St10 | Hanslick and Kruckenhauser, 2000 | F:TTGTGATCCTCCAGGGAGTT  R:GTGATTTCCAAACCCCATTC | 94ºC 5 min, (94ºC 20s, 52ºC 20s, 70ºC 20s) x36, 72ºC 2 min | 5μL Top Taq, 1μL of 10μM Primer, 2μL H_2_O, 2μL DNA | 2; 139, 141 |

**Table S2** Coancestry simulation for best fit relatedness estimator given actual allele frequencies, testing from unrelated to parent/offspring, with a population of 200.

|  | TrioML | Wang | LynchLi | LynchRd | Ritland | QuellerGt | DyadML | TrueValue |
| --- | --- | --- | --- | --- | --- | --- | --- | --- |
| Mean | 0.2679 | 0.23192 | 0.23243 | 0.2343 | 0.23297 | 0.21889 | 0.31447 | 0.23438 |
| Variance | 0.04932 | 0.14372 | 0.14508 | 0.12022 | 0.19408 | 0.12843 | 0.0638 | 0.04158 |
| MSE | 0.03926 | 0.10398 | 0.10484 | 0.08121 | 0.15312 | 0.09113 | 0.05109 |  |

LITERATURE CITED

da Silva A, Luikart G, Allainé D, Gautier P, Taberlet P, Pompanon F (2003) Isolation and characterization of microsatellites in European alpine marmots (*Marmota marmota*). Molecular Ecology Notes 3: 189-190.

Goossens B, Graziani L, Waits LP, Farand E, Magnolon S, Coulon J, Bel MC, Taberlet P, Allainé D (1998) Extra-pair paternity in the monogamous Alpine marmot revealed by nuclear DNA microsatellite analysis. Behavioral Ecology and Sociobiology 43: 281-288.

Hanslick S, Kruckenhauser L (2000) Microsatellite loci for two European sciurid species (*Marmota marmota, Spermophilus citellus*). Molecular Ecology 9: 2163-2165.

Klinkicht M (1993) Untersuchungen zum Paarungssystem des Alpenmurmeltiers, *Marmota m. marmota* mittels DNA fingerprinting. PhD thesis, University of Munich, Munich, Germany.

Kyle CJ, Karels TJ, Clark B, Strobeck C, Hik DS, Davis CS (2004) Isolation and characterization of microsatellite markers in hoary marmots (*Marmota caligata*). Molecular Ecology Notes 4: 749-751.

Stevens S, Coffin J, Strobeck C (1997) Microsatellite loci in the Columbian ground squirrels *Spermophilus columbianus*. Molecular Ecology 6: 493-495.


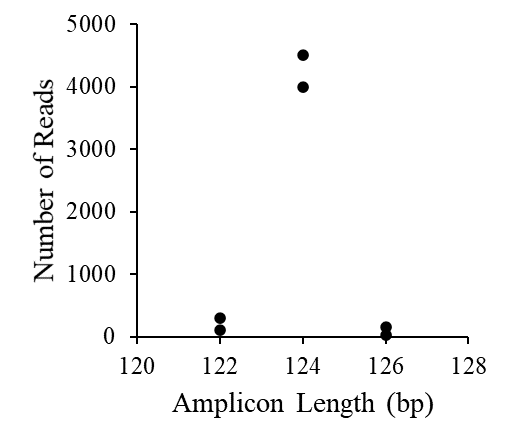


(c)


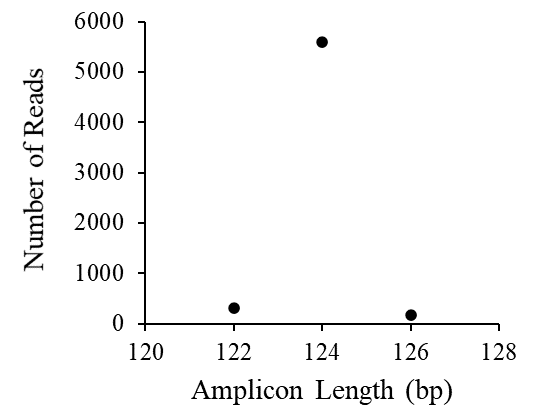


(a)


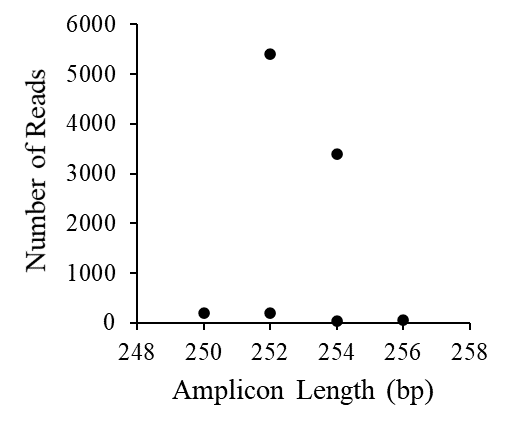


(b)

**Fig. S1** Representations of stereotypical peak patterns for (a) a homozygous individual for allele 124 (b) a heterozygous individual for alleles 252 and 254, and (c) a heterozygote individual with two different allele sequences (hidden homoplasy) at 124 bp. All patterns show low-copy stutter peaks two bases shorter and longer than the primary alleles.

(b)

(a)

**Fig. S2** Outputs from *StructureSelector* estimating the optimal number of genetic clusters with the mean ln Pr(*X*|*K*) using (a) HTAS genotypes from 11 loci and (b) length-based genotypes from six loci.


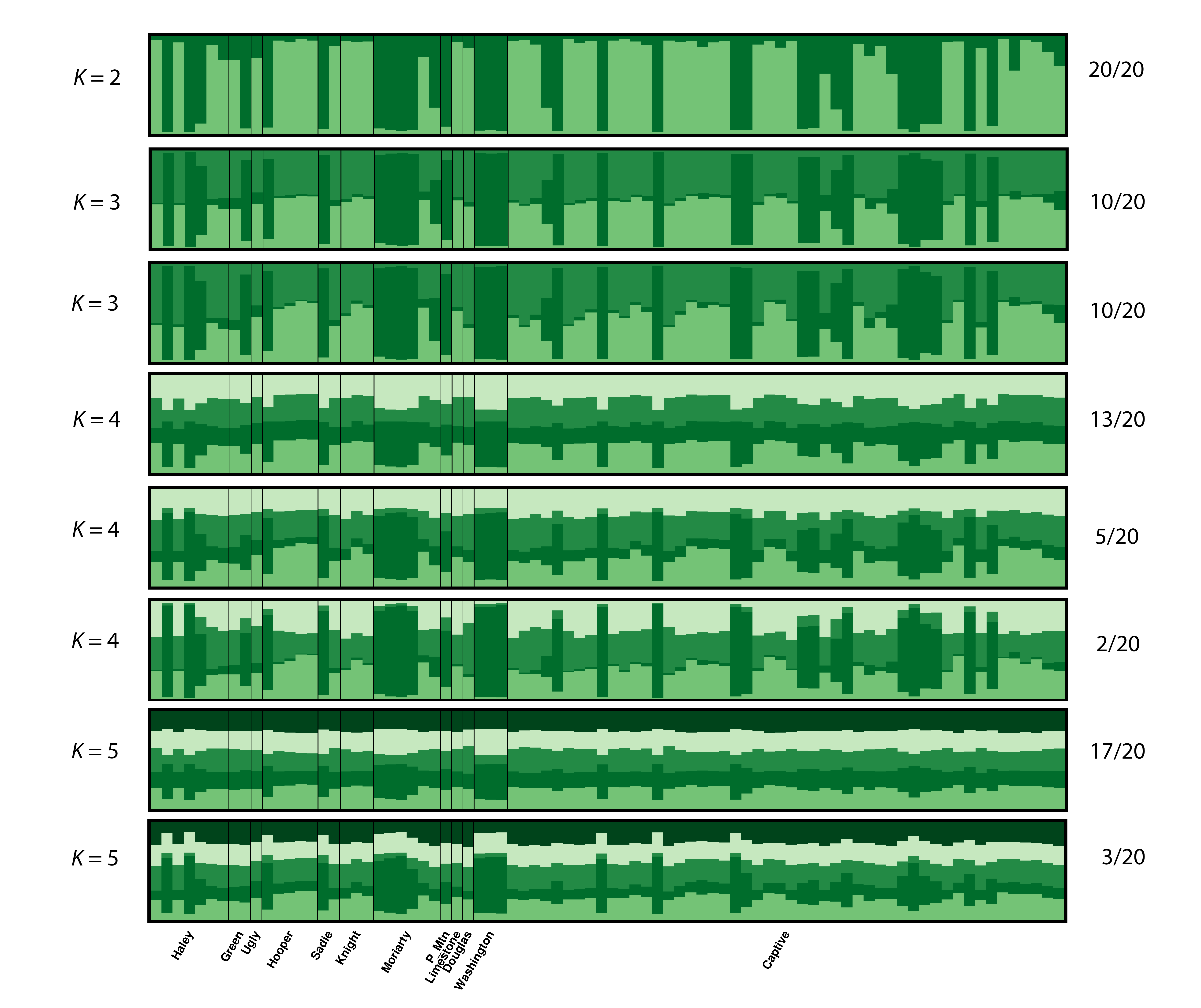


**Fig. S3** Membership probabilities among samples from wild (*n* = 32) and captive (*n* = 50) marmots across 20 STRUCTURE runs using HTAS genotypes from 11 loci.


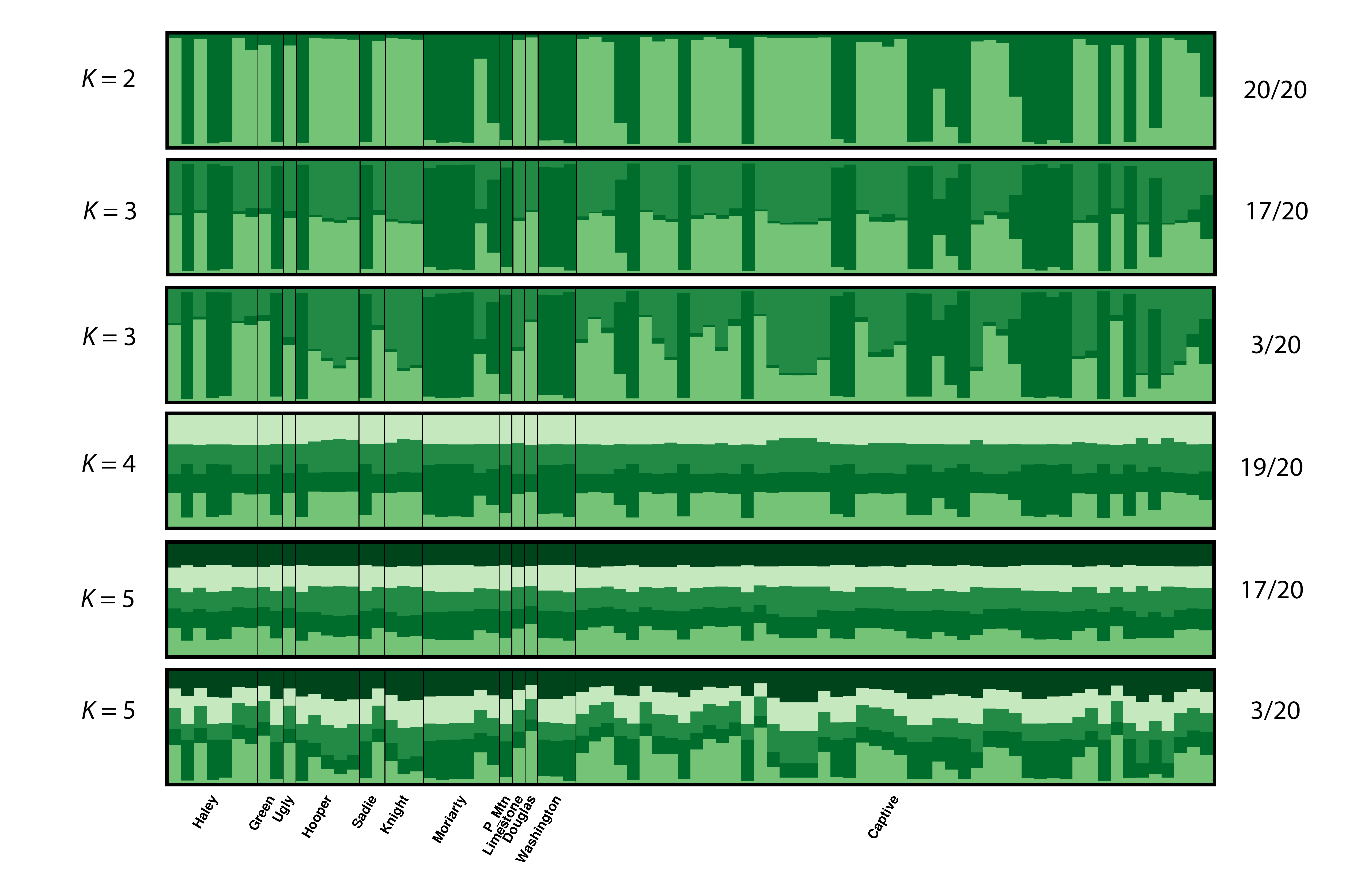


**Fig. S4** Membership probabilities among samples from wild (*n* = 32) and captive (*n* = 50) marmots across 20 STRUCTURE runs using length-based genotypes from six loci.
